# Mental health legislation in the Philippines: Philippine Mental Health Act

**DOI:** 10.1192/bji.2018.33

**Published:** 2019-08

**Authors:** John Lally, Rene M. Samaniego, John Tully

**Affiliations:** 1 Psychiatrist and Clinical Lecturer, Department of Psychosis Studies, Institute of Psychiatry, Psychology and Neuroscience, King's College London, UK. Email: john.lally@kcl.ac.uk; 2 Psychiatrist and Clinical Lecturer, Department of Psychiatry, Royal College of Surgeons in Ireland, Beaumont Hospital, Ireland; 3 Psychiatrist and Clinical Lecturer, Department of Psychiatry, School of Medicine and Medical Sciences, University College Dublin, St Vincent's University Hospital, Ireland; 4 Psychiatrist, Section of Psychiatry, Department of Neurosciences, Makati Medical Center, the Philippines; 5 Psychiatrist and Clinical Lecturer, Department of Forensic and Neurodevelopmental Sciences, Institute of Psychiatry, Psychology and Neuroscience, Kings College London, UK

**Keywords:** Psychiatry and law, low- and middle-income countries, Philippines, ethics

## Abstract

The first mental health act legislation in the history of the Philippines has been officially signed into law and was enacted as the Republic Act no. 11036 on 21 June 2018. It provides a rights-based mental health bill and a comprehensive framework for the implementation of optimal mental healthcare in the Philippines. We review the principles and provisions of the Mental Health Act of 2017 and the implications for mental healthcare in the Philippines.

‘Necessity is the most powerful divinity the world knows – it is the result of physical forces set in operation by ethical forces.’ (Rizal, 1912)

The Philippines is an autonomous republic located in the western Pacific, with a population of over 100 million people (Philippines Statistics Authority, 2016). Despite its size and its reputation for highly trained healthcare professionals, there is a paucity of epidemiological evidence for mental disorders in the Philippines. What is known, according to the 2010 census, is that 200 000 people were identified to have a disability due to mental disorder (Philippines Statistics Authority, 2010). This equated to 14% of a total of 1.4 million Filipinos with disabilities. The incidence of suicide has increased decade on decade, rising from 0.23 to 3.59 per 100 000 in males and from 0.12 to 1.09 per 100 000 in females between 1984 and 2005 (Redaniel *et al*, 2011).

Although the Philippine National Mental Health policy was enacted in 2001, mental health remains a poorly resourced sector of healthcare in the Philippines. Only 3–5% of the total health budget is spent on mental health, and the majority of this is spent on the operation and maintenance of psychiatric hospitals (WHO and Department of Health, 2006). The ratio of mental health workers to the population in the Philippines is low at 2–3 per 100 000 population (WHO-AIMS Report; WHO and Department of Health, 2006), with a little over 500 psychiatrists in practice. The majority of psychiatrists work in for-profit services or private practices, mainly in the major urban areas, especially in the capital region of Metro Manila (Samaniego, 2017).

Despite these problems, raised awareness of mental illness in recent times has led to increased governmental application and focus. Much credit for this is due to the Philippines Psychiatric Association (PPA). Founded in 1972, the PPA has worked tirelessly to promote mental health as a priority in the Philippines and it has advocated for the enactment of comprehensive mental health legislation into law in the form of a mental health act.

## Mental health legislation: the Philippine Mental Health Act (Republic Act no. 11036)

Proposed more than 3 years ago, the Philippine Mental Health Act was passed in the congress and senate in 2017 (Senate Bill No. 1354, 2017) and signed into law on 21 June 2018. Prior to this bill, the Philippines were one of a minority of countries with no mental health legislation. Clinicians lacked guidance on legal and ethical aspects of their practice, and patients' rights were not clearly defined – for example, the usual practice was for patients who lacked capacity to be ‘signed in’ by a next of kin. The passing of this bill is a major milestone in the history of psychiatry in the Philippines. The bill, the first in the country's history, provides a rights-based mental health legislation. It mandates for the provision of psychiatric, psychosocial and neurological services in all hospitals, and basic mental health services in community settings. Compulsory treatment is limited to hospital settings, and the Act does not provide for compulsory community treatment.

Under its provisions, the Philippine Mental Health Act protects the rights of patients as follows: ‘a right to freedom from discrimination, right to protection from torture, cruel, inhumane, and degrading treatment; right to aftercare and rehabilitation; right to be adequately informed about psychosocial and clinical assessments; right to participate in the treatment plan to be implemented; right to evidence-based or informed consent; right to confidentiality; and right to counsel, among others’.

The Act also incorporates rights for ‘concerned individuals’, incorporating patient relatives and mental health professionals. In this context, a mental health professional refers to a medical doctor, psychologist, nurse, social worker or any other appropriately-trained and qualified person with specific skills relevant to the provision of mental health services (section 4 of the Act). The Act highlights the need to provide psychosocial support to family members of the patient if required and, with informed patient consent, to include them in the planning of treatment for the patient.

It further recognises the role of mental health professionals, protecting their right to participate in mental health planning and development of services, and ensuring that they have a safe working environment, access to continuing education and autonomy in their own practice. Additionally, and with some foresight, the Act seeks to integrate mental health into the educational system by promoting mental health programmes in schools and other organisations.

## Principles of the Mental Health Act

In keeping with the United Nations (UN) resolution, the Principles for the Protection of Persons with Mental Illness and for the Improvement of Mental Health Care (United Nations, 1992), to which the Philippines is a signatory, the Philippines Mental Health Act of 2017 has several core principles including those covering the following areas:

### Definition of mental illness

According to the Act, ‘“Mental Health Condition” refers to a neurologic or psychiatric condition characterized by the existence of a recognizable, clinically-significant disturbance in an individual's cognition, emotional regulation, or behaviour that reflects a genetic or acquired dysfunction in the neurobiological, psychosocial, or developmental processes underlying mental functioning. The determination of whether a mental health condition exists shall be based on the best available scientific and medical evidence’. The new bill covers all mental disorders, inclusive of substance-related disorders, and involves active treatment of substance-related issues and preventive measures such as incorporation of psychoeducational topics in the education system, from elementary school onwards.

### Informed consent

The Act provides for ‘Free Prior Informed Consent’ or ‘Informed Consent’, referring to consent voluntarily given by a service user to a plan for treatment. A patient must ‘give prior informed consent before receiving treatment or care, including the right to withdraw such consent’. ‘All persons … shall be presumed to possess legal capacity for the purposes of this Act or any other applicable law, irrespective of the nature or effects of their mental health condition or disability’.

The Act makes provision to treatment without informed consent, ‘during psychiatric or neurologic emergencies, or when there is impairment or temporary loss of capacity on the part of a service user’. In such instances, ‘treatment, restraint or confinement, whether physical or chemical, may be administered or implemented pursuant to the following safeguards and conditions:’ service user's advance directives; only to the extent that such treatment or restraint is necessary; and only while a psychiatric or neurologic emergency, or impairment or temporary loss of capacity, exists or persists.

### Legal representatives and supported decision-making

Under the Act, any person subject to the Act may designate a person of legal age to act as his or her legal representative through a notarised document. This legal representative shall ‘provide the service user with support and help represent his or her interests; receive medical information about the service user in accordance with this Act; assist the service user *vis-á-vis* the exercise of any right provided under this Act; and be consulted with respect to any treatment or therapy received by the service user’. If a legal representative is not chosen, other persons can act as the legal representative, including the spouse, non-minor children and either parent by mutual consent, if the service user is a minor. A person subject to the Act may also designate up to three persons or ‘supporters’, including the service user's legal representative, for the purposes of supported decision-making.

### Other considerations

The Act also addresses protection of the rights of persons with mental disorders in mental health facilities, protection of minors, provision of resources for mental health facilities, role of community and culture, review mechanisms providing for the protection of the rights of offenders with mental disorders, procedural safeguards protecting the rights of persons with mental disorders, protection of confidentiality, and standards of care and treatment including involuntary admission and consent to treatment. The Act does not mention specific types of treatment, such as electroconvulsive therapy, but its section on ‘Quality of Mental Health Services’ states that treatments must be based on medical and scientific research findings, responsive to individual and cultural needs, provided in the least restrictive setting and provided by mental health professionals and workers in a manner that ensures accountability.

A pertinent issue facing many jurisdictions is whether their mental health legislation is compatible with the UN Convention on the Rights of Persons with Disabilities, including those with ‘mental impairments’. The UN regional authorities were involved in the process of drafting and further revisions of the Act, along with other important stakeholders such as patients and their families, the Commission on Human Rights and the World Association for Psychosocial Rehabilitation.

A further consideration is the practical implementation and impact of the new Act, and how this will be monitored. The PPA is currently in the process of implementing a set of rules and regulations, which are due to be completed within 90 days from the signing of the Act. A ‘Healthy Mind’ summit in October 2018 involved all of the stakeholders in a joined review of the law, with the aim of identifying practical challenges and providing a critical perspective.

## Conclusion

Mental healthcare remains an under-resourced and neglected aspect of healthcare in the Philippines. Until now, the country has lacked a formal structure in which to enshrine the rights of those people with mental illness, their families, and the rights and responsibilities of mental health professionals and government in relation to mental health. The Philippine Mental Health Act of 2017 has created an environment for the organisation and provision of hospital- and community-based mental healthcare in the Philippines, while providing specific legislative checks to ensure the rights of patients receiving mental healthcare and treatment are protected.

The Philippine Mental Health Act of 2017 is therefore a major step forward for mental health in the Philippines and a milestone for psychiatry in the country. Under the direction of the PPA, willing governmental support along with the driving enthusiasm of mental health professionals, patient groups and family members have led to the first comprehensive Mental Health Act legislation in the Philippines. Significant logistical challenges remain in the successful implementation of this legislation, but the Act is a significant step with the provision to comprehensively address, at a national and local level, the mental health needs of the population.
